# Ectopic Gestation Sac Opening into Rectum

**Published:** 1912-09

**Authors:** 


Ectopic Gestation Sac Opening into Rectum.—Brickner, *Am. Jour. of Obstet.*, Feb. 1911, describes the case of a woman who, having had one child at term and missing a period by four weeks, introduced a bougie into the uterus, apparently miscarrying and requiring currettement. The case pursued a somewhat septic course for two weeks when bleeding from the bowel began and a fish bone was found lodged in the rectum. The bleeding and septic manifestations continued, with a mass to the right of the uterus. This was found, on operation to be a gestation sac, the right tube having opened up the broad ligament and ultimately discharged into the rectum. In all, the gestation sac, four inches of bowel, the appendix which was applied to the sac, and the uterus above the cervix, had to be resected. Barring a temporary fæcal fistula, the patient made a good recovery.

